# Autophagy Limits Inflammasome During *Chlamydia pneumoniae* Infection

**DOI:** 10.3389/fimmu.2019.00754

**Published:** 2019-04-12

**Authors:** Timothy R. Crother, Rebecca A. Porritt, Jargalsaikhan Dagvadorj, Gantsetseg Tumurkhuu, Anatoly V. Slepenkin, Ellena M. Peterson, Shuang Chen, Kenichi Shimada, Moshe Arditi

**Affiliations:** ^1^Division of Pediatric Infectious Diseases and Immunology, Department of Pediatrics, and Infectious and Immunological Diseases Research Center, Department of Biomedical Sciences, Cedars-Sinai Medical Center, Los Angeles, CA, United States; ^2^Department of Pediatrics, David Geffen School of Medicine at UCLA, Los Angeles, CA, United States; ^3^Department of Microbiology and Molecular Genetics, University of California, Irvine, Irvine, CA, United States; ^4^Department of Pathology, University of California, Irvine, Irvine, CA, United States

**Keywords:** autophagy, *Chlamydia pneumoniae*, inflammasome, macrophages, IL-1β

## Abstract

Autophagy can either antagonize or promote intracellular bacterial growth, depending on the pathogen. Here, we investigated the role of autophagy during a pulmonary infection with the obligate intracellular pathogen, *Chlamydia pneumoniae* (CP). In mouse embryonic fibroblasts (MEFs) or macrophages, deficiency of autophagy pathway components led to enhanced CP replication, suggesting that autophagy exerts a bactericidal role. However, *in vivo*, mice with myeloid-specific deletion of the autophagic protein ATG16L1 suffered increased mortality during CP infection, neutrophilia, and increased inflammasome activation despite no change in bacterial burden. Induction of autophagy led to reduced CP replication *in vitro*, but impaired survival in CP-infected mice, associated with an initial reduction in IL-1β production, followed by enhanced neutrophil recruitment, defective CP clearance, and later inflammasome activation and IL-1β production, which drove the resulting mortality. Taken together, our data suggest that a delicate interplay exists between autophagy and inflammasome activation in determining the outcome of CP infection, perturbation of which can result in inflammatory pathology or unrestricted bacterial growth.

## Introduction

Autophagy, the process by which cellular material is recycled (1), has been increasingly found to play a central role in diverse diseases and pathologies (2). One key role for autophagy is the removal of damaged mitochondria (3), termed mitophagy, which in turn can modulate inflammatory processes by dampening activation of the nucleotide binding domain and leucine-rich repeat (NLR) pyrin domain containing 3 (NLRP3) inflammasome (4–8). Thus, loss of autophagic function can lead to enhanced inflammation via greater NLRP3 inflammasome activation and secretion of IL-1β. Release of this pro-inflammatory cytokine is tightly regulated, because although IL-1β is critical in controlling many microbial infections, over production can lead to enhanced pathology and chronic inflammation (9, 10).

In addition to regulating cytokine production, autophagy can play a direct role in microbial clearance in a process known as xenophagy (11). This is particularly important for intracellular bacteria such as *Salmonella typhimurium*, group A *Streptococcus*, and *Mycobacterium tuberculosis*. In these cases, the autophagic phagophore forms around the invading bacteria, and the resulting autophagosome is targeted for fusion with the lysosome (12–14). Bacteria can escape this eradication by actively preventing lysosomal fusion (15, 16), or autophagy induction and/or elongation (17). A related process that uses many components of the autophagosome machinery, termed LC3 associated phagocytosis (LAP), targets the production of reactive oxygen species in the phagosome lumen to directly kill bacteria such as *S. typhimurium* and *Burkholderia pseudomallei* (18, 19). Finally, some bacterial agents can co-opt components of the autophagosomal machinery in order to survive and replicate (20, 21).

CP is an obligate intracellular bacterium that mainly infects macrophages and neutrophils, and exists in an inclusion inside the cell (22). While CP infection is thought to be responsible for up to 10–20% of cases of community-acquired pneumonia (23), it has also been associated with many chronic inflammatory diseases including atherosclerosis, Alzheimer's disease, and asthma (24). CP have a unique lifecycle whereby they exist as an infectious elementary body (EB), which enters the host cell and forms an inclusion (22). Once inside, it changes into the metabolically active reticulate body (RB) and replicates inside the inclusion (22). Eventually RBs revert to the EB state and burst forth from the cell (22). While CP inclusions do not fuse with the lysosome, lysosomal maturation seems to be a requirement for CP growth (25), raising the possibility that the autophagy pathway plays a role in either preventing or facilitating CP infection.

In this study we found blocking autophagy led to increased CP growth in both macrophages and mouse embryonic fibroblasts. *In vivo*, loss of the autophagy elongation component ATG16L1 specifically in myeloid cells led to increased mortality in response to CP infection, characterized by greater numbers of neutrophils and dendritic cells, but no change in the CP burden in the lungs. This was accompanied by an increase in inflammasome-active macrophages and IL-1β production. While induction of autophagy in macrophages led to reduced CP growth *in vitro, in vivo* treatment with rapamycin led to increased mortality of infected mice, likely due to initial impairment in IL-1β production resulting in increased bacterial load.

## Materials and Methods

### Mice and Cell Lines

C57BL/6 (wild-type [WT]) mice were purchased from Jackson Labs (Bar Harbor, ME) and then bred in the Cedars-Sinai Medical Center Comparative Medicine facility. *Atg16l1*^*fl*/*fl*^ *Lysm*^*cre*^ mice were obtained from Dr. David Shih, Cedars-Sinai medical Center (26). *Atg16l1*^*fl*/*fl*^ mice were used as littermate controls. Eight- to ten-weeks-old female mice were used in all experiments in this study. *WT, Atg16l1*^+/−^, and *Atg5*^−/−^ mouse embryonic fibroblasts (MEF)s were obtained from Dr. Jae Jung (University of Southern California) (27) and grown in DMEM media containing 10% FBS. In some cases, 1 μg/ml cycloheximide (Sigma Aldrich, St. Louis MO), MEM amino acids (Sigma Aldrich, St. Louis MO), or Na-pyruvate (Sigma Aldrich, St. Louis MO) were added to the media. *Atg5*^*fl*/*fl*^ *Lysm*^*cre*^ and *Atg5*^*fl*/*fl*^ bone marrow was provided by Dr. Herbert W. Virgin (Washington University, Saint Louis).

### Ethics Statement

All experiments were conducted according to Cedars-Sinai Medical Center Institutional Animal Care and Use Committee (IACUC) guidelines (IACUC# 8314).

### CP Infection Model

*Chlamydia pneumoniae* CM-1 (American Type Culture Collection, Manassas, VA) was propagated in HEp-2 cells, as previously described (28). Both HEp-2 cells and *C. pneumoniae* aliquots were confirmed as being Mycoplasma- free by PCR. Mice were infected with either 1 × 10^6^ or 2.5 × 10^6^ inclusion forming units (IFU) *C. pneumoniae* by inoculating intratracheally. Bronchoalveolar lavage fluid (BALF) was collected by injecting 0.5 ml PBS containing 5 mM EDTA. BALF was separated into supernatant and cells by centrifugation. Supernatant was used for cytokine and chemokine measurements, and cells were analyzed by Flow Cytometry. In some experiments, mice were injected with rapamycin (3 mg/kg; ThermoFisher Scientific, MA, USA) i.p., every other day until sacrifice. The first injection was given 6 h prior to infection.

### CP IFU Determination

HEp-2 cells were infected with *C. pneumoniae* in lung specimens for bacterial burden quantification, as described previously (29). Briefly, HEp-2 cells were incubated with diluted lung suspensions or cell lysates in the presence of RPMI 1640 medium containing 1 μg/ml cycloheximide. Centrifugation was performed for 1 h at 800 x g, and cells were placed in an incubator (37°C, 0.5% CO_2_). After 72 h of culture, cells were washed with PBS, fixed with methanol, and stained with FITC-conjugated anti-Chlamydia genus-specific mAb (Pathfinder Chlamydia Culture Confirmation System; Bio-Rad, Hercules, CA), according to the manufacturer's instructions. *C. pneumoniae* inclusions in cells were counted by fluorescence microscopic analysis. For *in vitro* inclusion quantification, infected cells were directly assessed using the Pathfinder Chlamydia Culture Confirmation System as above.

### Flow Cytometry

BALF cellular samples were used for Flow cytometry analysis. Cells were filtered through a 70 μm cell strainer (BD Bio-sciences, San Jose, CA). Erythrocytes were obviated by RBC Lysis Buffer (eBioscience, San Diego, CA). Single cells were stained with the following antibodies (Ab)s (Tonbo Biosciences, San Diego, CA): F4/80 Ab (BM8), CD11c Ab (N418), Ly6G Ab (1A8), CD11b Ab (M1/70), and CD3 Ab (145-2C11). For BALF single-cell differentials, cell types were identified as neutrophils (CD11c^−^ CD11b^+^ Ly6G^+^), alveolar macrophages (AM)s (CD11b^+^ CD11c^+^ F4/80^+^), dendritic cells (CD11c^+^ CD11b^+^ F4/80^−^), and T cells (CD3^+^). Flow cytometric analysis was performed using a CyAn flow cytometer (Beckman Coulter) and analyzed using FlowJo software (FlowJo LLC, Ashland, OR).

### ELISA

The concentration of cytokine in BALF was determined using an OptEIA Mouse IL-6 ELISA Set (BD Biosciences) and Mouse IL-12p40, Mouse IFN-γ ELISA Kit (eBioscience), and DuoSet Mouse MIP-2 (CXCL2) and Mouse KC (CXCL1) (R&D Systems, Minneapolis, MN). These assays were performed according to the manufacturers' instructions. IL-1β in mouse BALF samples was measured using the U-PLEX Mouse IL-1β Assay (Meso Scale Diagnostics, Rockville, Maryland) per the manufacturer's instructions. The samples were read and analyzed by MSD QuickPlex SQ120 instrumentation and Workbench 4.0 Software (Meso Scale Diagnostics, Rockville, Maryland).

### Pulmonary Inflammation Scoring

Lungs were fixed in formalin, paraffin embedded and sectioned. Lung sections were stained with hematoxylin and eosin. Inflammatory scoring was performed by a blinded pathologist, scoring 1–4 as previously described (30).

### Immunofluorescence

Caspase-1 activity was detected by Fluorochrome-labeled inhibitors of caspases assay (FLICA) from Immunochemistry Technologies (Bloomington, MN) per the manufacturer's instructions for either frozen sections or live tissue culture. For Immunofluorescence staining for frozen sections, fixing and antigen blocking were performed using immunoglobulin from the species of the secondary antibodies. Next, the sections were incubated with primary antibodies for 2 h RT, followed by incubation with appropriate secondary antibodies conjugated with Fluorescent dyes. For identification of FLICA+ cells, anti-F4/80 (BM8) antibody and anti CD11c (N418) (eBioscience) were used.

### Bone Marrow Derived Macrophages (BMDM) and Isolation of Peritoneal Macrophages

BMDM: Femora and tibiae of 8–10 week old C57BL/6 were flushed with RPMI 1640 medium. Bone marrow cells were cultured in RPMI 1640 medium containing 10% FBS and 15% L929 cell conditioned medium with 2 media changes. BMDM were harvested on day 7. Elicited peritoneal macrophages: 1 ml thioglycollate media was injected i.p., in mice. 3–4 days later peritoneal cells were obtained by syringe and plated. Twenty four hour later, non-adherent cells were washed away.

### Statistics

Data are presented as mean ± SEM. For survival studies, significance was evaluated by the log-rank test. For comparisons between two groups, statistical significance was evaluated using Student's two-tailed unpaired *t*-test. For non-normally distributed data, unpaired Mann–Whitney test was used. For experiments in which more than two samples were analyzed, one-way ANOVA with the Tukey *post-hoc* test were used to assess statistical significance between groups. *p* < 0.05 was considered significant.

## Results

### Autophagy Regulates *Chlamydia pneumoniae* Inclusion Formation

**Figure 1:**
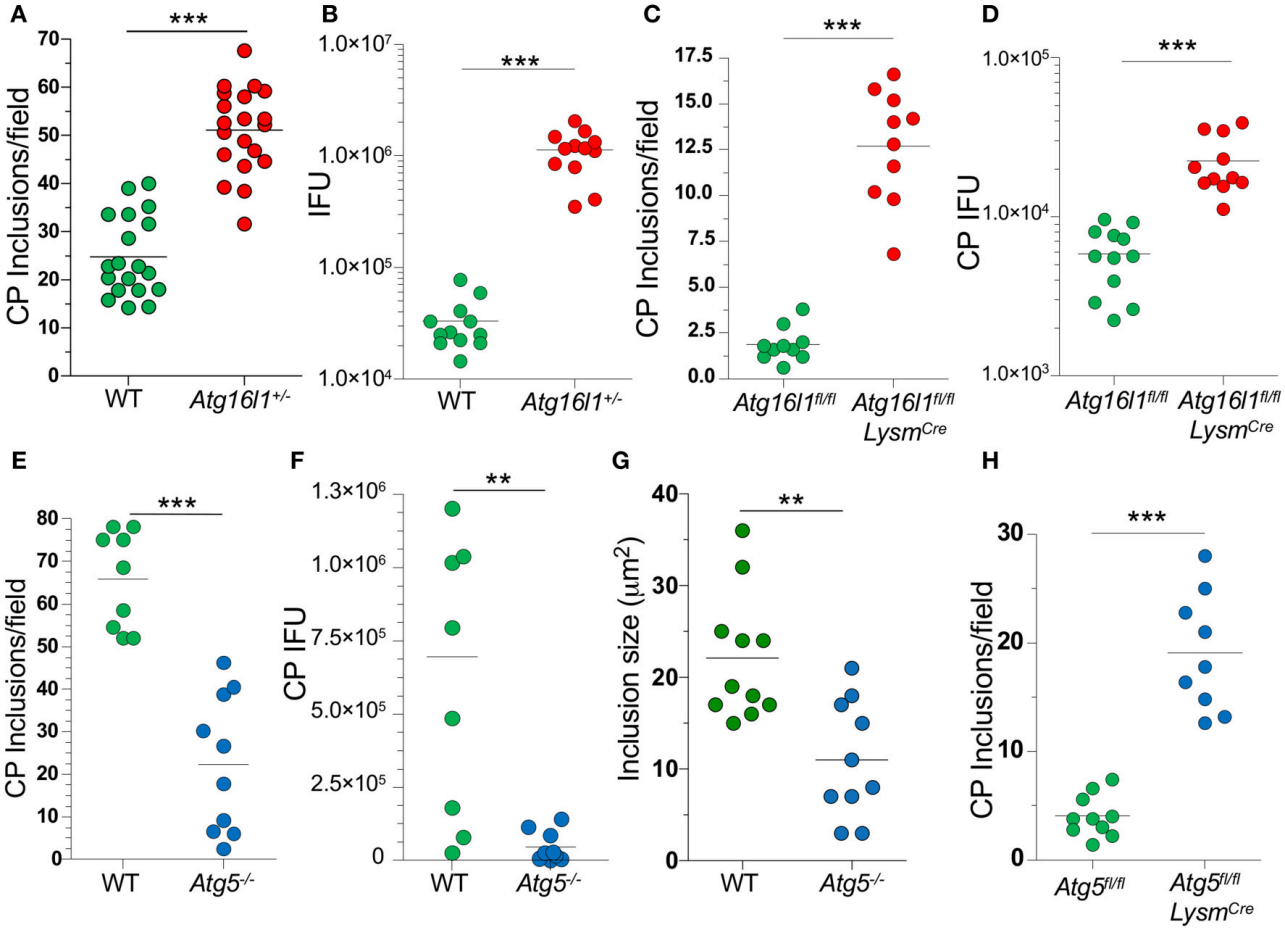
Autophagy plays a role during CP growth. **(A,B)** WT and *Atg16l1*^+/−^ MEFs were infected with CP (MOI 10) and 68 h later assessed for inclusions **(A)** and IFUs **(B)**. **(C,D)** *Atg16l1*^*fl*/*fl*^ and *Atg16l1*^*fl*/*fl*^ *Lysm*^*cre*^ BMDM were infected with CP (MOI 1) and 68 h later assessed for inclusions **(C)** and IFUs **(D)**. **(E–G)** WT and *Atg5*^−/−^ MEFs were infected with CP (MOI 10) and 68 h later assessed for inclusions **(E)**, IFUs **(F)**, and inclusion size **(G)**. **(H)** *Atg5*^*fl*/*fl*^ and *Atg5*^*fl*/*fl*^ *Lysm*^*cre*^ BMDM were infected with CP (MOI 1) and 68 h later assessed for inclusions **(C)**. All panels are representative of three experiments. Significance was assessed by students *t*-test. ^**^p < 0.01, ^***^p < 0.001.

In order to understand what role autophagy might play during CP growth, we infected MEFs with CP and observed that 48 h after infection, most of the LC3 was lipidated (LC3-II), suggestion an increase in autophagy (Supplemental Figure 1A). We next infected *Atg16l1*^+/−^ MEFs and observed a significant increase in the number of inclusions present when compared with WT MEFS (Figure 1A). ATG16L1, along with ATG5 and ATG12, make up the elongation complex of autophagosome formation (31). By gross observation, the CP inclusions in *Atg16l1*^+/−^ MEFs were often much larger than those in WT MEFS (Supplemental Figure 1). In addition to an increase in inclusion numbers, *Atg16l1*^+/−^ cells developed a greater number of CP progeny as measured by inclusion forming units (IFU) (Figure 1B). We observed a similar increase in inclusion numbers as well as progeny in CP infected bone marrow derived macrophages from At*g16l1*^*fl*/*fl*^ *Lysm*^*Cre*^ mice (Figures 1C,D). Surprisingly, we observed the opposite phenotype when cells were deficient in another autophagy component, Atg5, as CP-infected *Atg5*^−/−^ MEFs with CP had significantly fewer inclusions and progeny than WT cells (Figures 1E,F). Additionally, the CP inclusions observed in *Atg5*^−/−^ MEFs were significantly smaller than those in WT MEFs (Figure 1G, Supplemental Figure 1B). This discrepancy between *Atg16l1*^+/−^ MEFs and *Atg5*^−/−^ MEFs was surprising as both ATG16L1 and ATG5 participate in the same autophagic complex, along with ATG12. However, CP-infected *Atg5*^−/−^ macrophages obtained from *Atg5*^*fl*/*fl*^ *Lysm*^*Cre*^ mice displayed higher inclusion numbers that WT macrophages (Figure 1H), similar to macrophages lacking ATG16L1. Thus, while it was clear that autophagic machinery played a role in CP growth in both MEFs and macrophages, the effect of loss of pathway components differed between cell types.

### *Atg5^−/−^* and *Atg16l1^+/−^* MEFs Have Altered Growth Rates Which Affects CP Inclusion Formation

**Figure 2:**
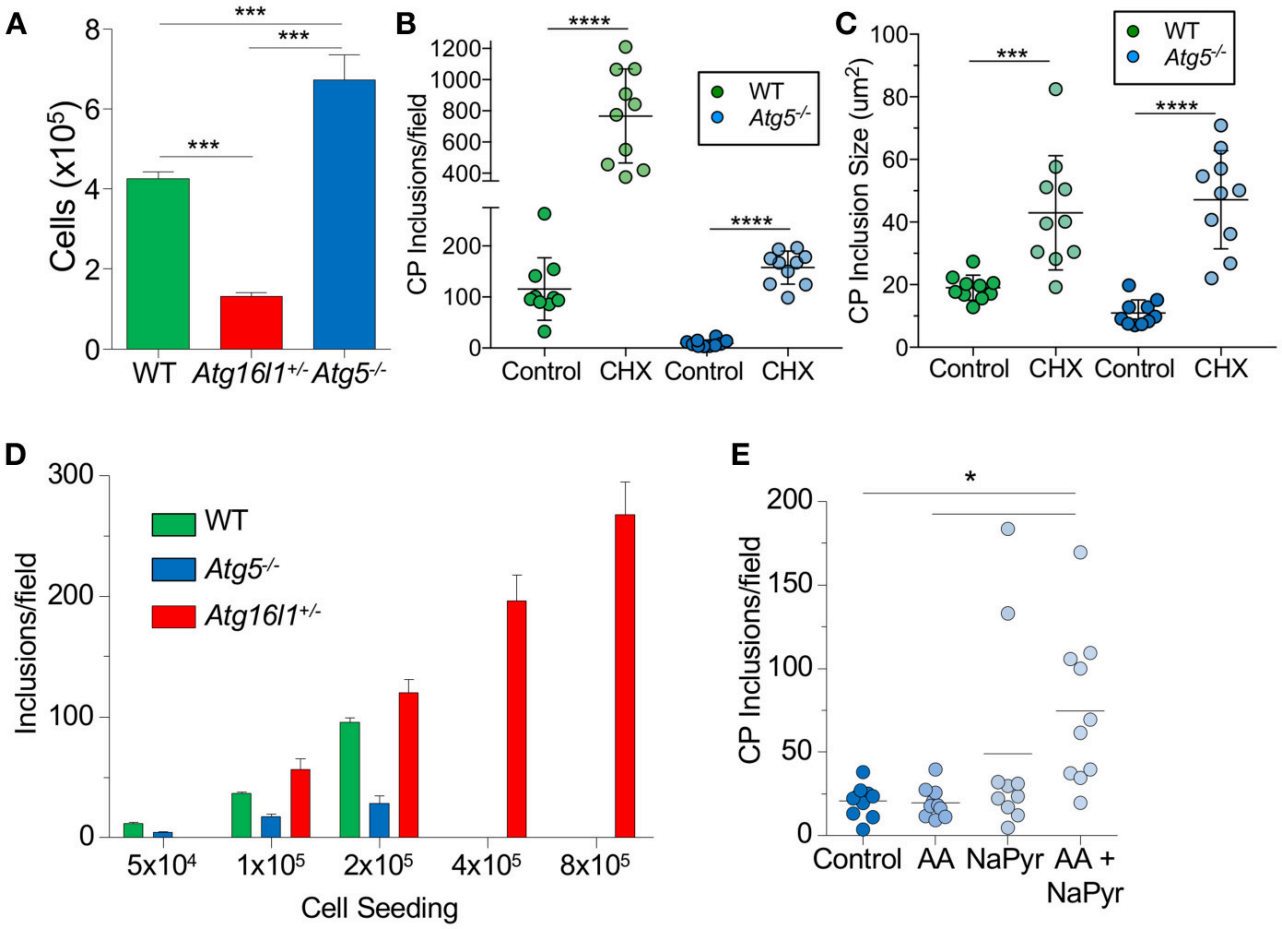
CP growth requires adequate nutrition available to the host. **(A)** WT, *Atg16l1*^+/−^, and *Atg5*^−/−^ MEFs were seeded at 5 × 10^4^ in a 24-well plate. Total cell numbers were counted after 3 days. **(B,C)** *Atg5*^−/−^ MEFs were infected with CP (MOI 10) and treated with 1 μg/ml cycloheximide. Inclusions were counted 68 h post infection **(B)**, as was inclusion size **(C)**. **(D)** WT, *Atg16l1*^+/−^, and *Atg5*^−/−^ MEFs were seeded at the indicated amounts in a 24-well plate and infected with CP (MOI 10). Inclusions were counted 68 h post infection. **(E)** *Atg5*^−/−^ MEFs were infected with CP (MOI 10) and the media was supplemented with either MEM amino acids (AA), Na-pyruvate (NaPyr), or both. Inclusions were counted 68 h post infection. All panels are representative of three experiments. Data are mean ± SEM. Significance was assessed by students *t*-test **(B,C)** or one-way ANOVA **(A,E)**. ^*^p < 0.05, ^***^p < 0.001, ^****^ *p* < 0.0001.

We next sought to understand why loss of ATG5 and ATG16L1 had differential effects on CP infection in MEFs. We observed that *Atg16l1*^+/−^ MEFs divided much more slowly than WT MEFs, while *Atg5*^−/−^ MEFs grew much more quickly (Figure 2A). We therefore suspected that the difference in CP inclusion formation between *Atg16l1*^+/−^ MEFs and *Atg5*^−/−^ MEFs may be due to cell growth rates and perhaps nutritional limits. Indeed, inhibiting cell growth with cycloheximide, which has also been reported to affect autophagy (32), resulted in a significant increase in the number and size of inclusions in *Atg5*^−/−^ MEFs (Figures 2B,C). An increase was also observed in WT MEFs treated with cycloheximide. We therefore reasoned that in nutrient-replete conditions, CP inclusion formation may be affected by differential growth rates. To test this, we seeded tissue culture plates with various starting numbers of MEFs and infected them with the same CP multiplicity of infection. Regardless of the starting cell number, *Atg16l1*^+/−^ MEFs were able to support high numbers of CP inclusions (Figure 2D). However, *Atg5*^−/−^ MEFs could only support CP inclusion growth at the lowest cell seeding density, and no inclusions were observed at higher starting cell numbers (Figure 2D). Similarly, while WT MEFs had greater numbers of inclusions than *Atg5*^−/−^ MEFs at lower seeding density, and they failed to support CP inclusion growth when seeded at high density (Figure 2D). Finally, when *Atg5*^−/−^ MEFs were supplied with additional amino acid supplement and sodium pyruvate, greater numbers of inclusions were observed (Figure 2E). Taken together, these data indicate that differential growth rates and subsequent depletion of nutrients underlies the difference in CP inclusion formation in *Atg16l1*^+/−^ and *Atg5*^−/−^ MEFs, and in macrophages, where there is little cell growth, loss of ATG5 and ATG16L1 both lead to increased numbers of CP inclusions and progeny, suggesting that autophagy plays a role in limiting CP growth.

### Autophagy Deficient Mice Are Susceptible to CP Infection *in vivo*

**Figure 3:**
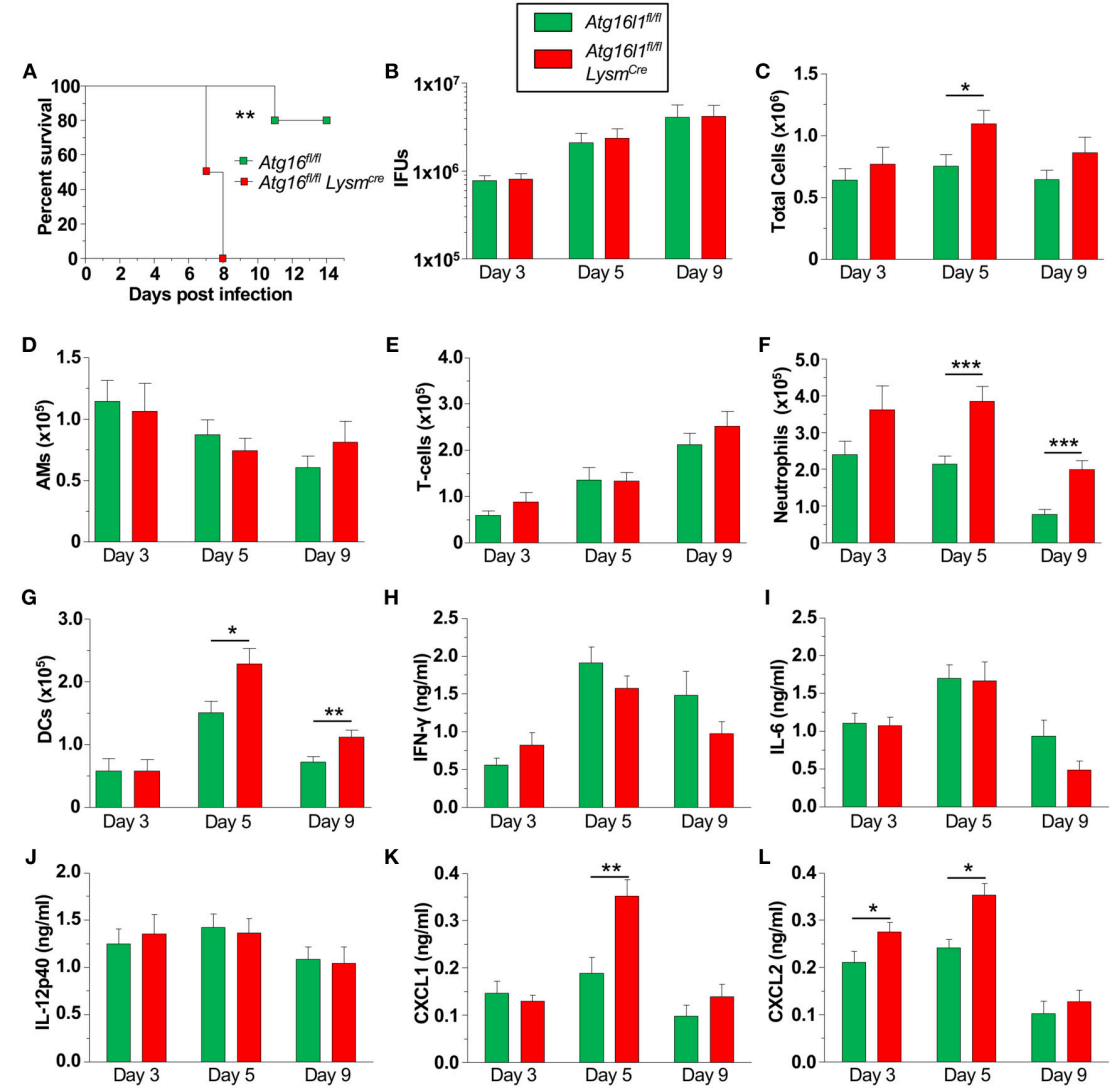
Loss of autophagy in myeloid cells leads to neutrophilia and mortality during CP infection. **(A)** *Atg16l1*^*fl*/*fl*^ and *Atg16l1*^*fl*/*fl*^ *Lysm*^*cre*^ mice were infected with 2.5 × 10^6^ IFU CP i.t. and mortality was assessed (*n* = 5–6). **(B–L)** *Atg16l1*^*fl*/*fl*^ and *Atg16l1*^*fl*/*fl*^ *Lysm*^*cre*^ mice were infected with 1.0 × 10^6^ IFU CP i.t. and mice were sacrificed 3, 5, and 9 days after infection (*n* = 9–13, pooled from two experiments). **(B)** Bacterial burden in the lungs. **(C)** Total cells in the BALF. **(D)** BALF Alveolar macrophages. **(E)** BALF T-cells. **(F)** BALF neutrophils. **(G)** BALF DCs. **(H)** BALF IFN-γ. **(I)** BALF IL-6. **(J)** BALF IL-12p40. **(K)** BALF CXCL1. **(L)** BALF CXCL2. Data are mean ± SEM. Significance was assessed by students *t*-test. Survival curve was compared using the Log-Rank (Mantel-Cox) Test. ^*^p < 0.05, ^**^p < 0.01, ^***^p < 0.001.

We next investigated how the loss of autophagy in phagocytic cells [macrophages (Supplemental Figure 2A), neutrophils, dendritic cells (DC)s etc.] would affect CP infection and clearance in mice. We found that *Atg16l1*^*fl*/*fl*^ *Lysm*^*Cre*^ mice were significantly more susceptible to CP infection than littermate *Atg16l1*^*fl*/*fl*^ mice, displaying 100% lethality within 8 days of infection (Figure 3A). In order to investigate the course of infection and immune responses, we infected *Atg16l1*^*fl*/*fl*^ *Lysm*^*Cre*^ and littermate *Atg16l1*^*fl*/*fl*^ controls with a lower inclusion forming unit (IFU) dose of CP. To our surprise, despite the differences observed in mortality, the bacterial burden was equivalent in the lungs of mice with myeloid autophagy deficiency and controls (Figure 3B). Bronchoalveolar lavage fluid (BALF) analysis revealed an increase in the number of cells in *Atg16l1*^*fl*/*fl*^ *Lysm*^*Cre*^ lungs 5 days after infection that resolved by day 9 (Figure 3C). While there were no differences in the numbers of AMs and T-cells between genotypes (Figures 3D,E), flow cytometry revealed a significant increase in both neutrophils and dendritic cells in the BALF of *Atg16l1*^*fl*/*fl*^ *Lysm*^*Cre*^ mice 5 and 9 days after infection (Figures 3F,G, Supplemental Figure 2B). Autophagy deficiency did not alter the levels of the critical pro-inflammatory cytokines required for normal immune responses to CP: IFN-γ, IL-6, or IL-12p40 (Figures 3H–J). However, in support of our finding of increased neutrophils, neutrophilic chemokines CXCL1 and CXCL2 were elevated in BALF from *Atg16l1*^*fl*/*fl*^ *Lysm*^*Cre*^ mice compared with controls (Figures 3K, L). Despite the increase in neutrophils and DCs, we did not observe a significant difference in gross pathology in the lungs by H&E between the conditional knockout and control mice (Supplemental Figure 2C). Thus, loss of autophagy in myeloid cells led to increased lung infiltration by neutrophils and DCs in the setting of CP infection and impaired survival but does not affect CP burden.

### Autophagy Deficiency Leads to Increased Pulmonary Inflammasome Activity and IL-1β Production in Response to CP Infection

**Figure 4:**
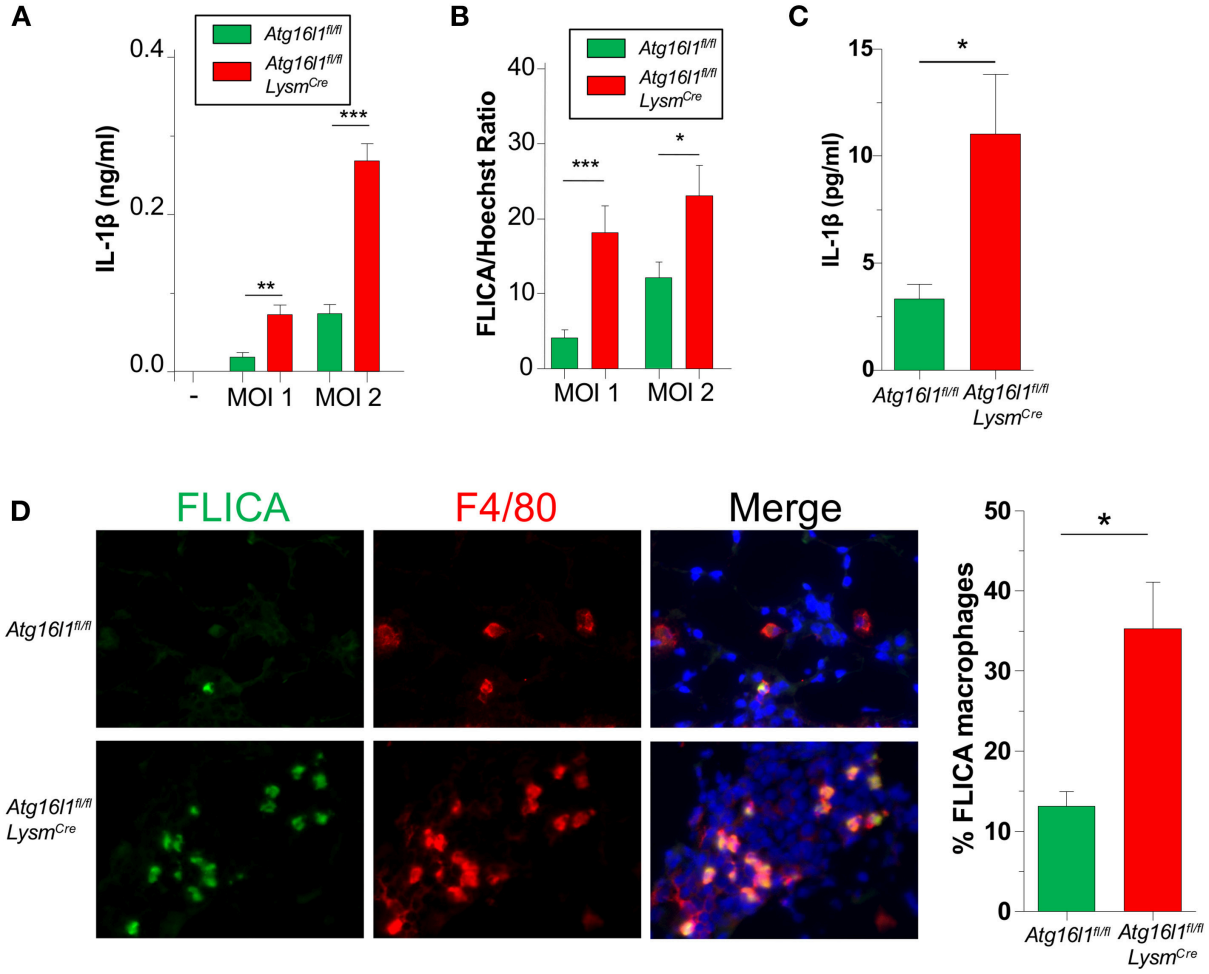
Loss of autophagy in myeloid cells leads to an increase in inflammasome activation and IL-1β during CP infection. **(A,B)** *Atg16l1*^*fl*/*fl*^ and *Atg16l1*^*fl*/*fl*^ *Lysm*^*cre*^ peritoneal macrophages were infected with CP at indicated MOI and **(A)** IL-1β was measured in the supernatants by ELISA 20 h later. **(B)** % FLICA positive cells. **(C)** IL-1β in BALF 1 day after CP infection in *Atg16l1*^*fl*/*fl*^ and *Atg16l1*^*fl*/*fl*^ *Lysm*^*cre*^ mice (*n* = 9–11 pooled from two separate experiments). **(D)** Caspase-1 activity measured by FLICA in frozen lung sections 1 day after CP infection in *Atg16l1*^*fl*/*fl*^ and *Atg16l1*^*fl*/*fl*^ *Lysm*^*cre*^ mice (*n* = 7). Data are mean ± SEM. Significance was assessed by students *t*-test. ^*^p < 0.05, ^**^p < 0.01, ^***^p < 0.001.

We previously showed that IL-1β production via the NLRP3 inflammasome is critically required to control CP growth to successfully resolve the infection in mice (33). Furthermore, autophagy and mitophagy are known to negatively regulate the NLRP3 inflammasome and subsequent IL-1β secretion (5–7). Indeed, *Atg16l1*^*fl*/*fl*^ *Lysm*^*Cre*^ macrophages produced more IL-1β than controls in response to CP infection (Figure 4A), which correlated with increased Caspase-1 activity (Figure 4B, Supplemental Figure 3A).

We next assessed the effect of autophagy deficiency on IL-1 β production *in vivo*. *Atg16l1*^*fl*/*fl*^ *Lysm*^*Cre*^ mice had significantly more IL-1β in BALF 1 day after infection compared with control animals (Figure 4C), corroborating out *in vitro* data. Finally, we also assessed Caspase-1 activity in the lungs of CP-infected mice 1 day post infection. Confirming our *in vitro* results, lung sections from *Atg16l1*^*fl*/*fl*^ *Lysm*^*Cre*^ showed significantly more FLICA positive macrophages compared with littermate controls (Figure 4D, Supplemental Figure 3B). Interestingly, the FLICA positive macrophages (F4/80^+^) that we observed were not CD11c^+^ (Supplemental Figure 3C), suggesting that these were not alveolar macrophages (AMs), but instead of a different origin, such as interstitial macrophages or newly recruited monocytes. Additionally, while it does not completely rule out other cell types secreting IL-1β in this model, we did not observe any non F4/80^+^ FLICA^+^ cells.

### Induction of Autophagy Limits CP Growth *in vitro* but Not *in vivo*

**Figure 5:**
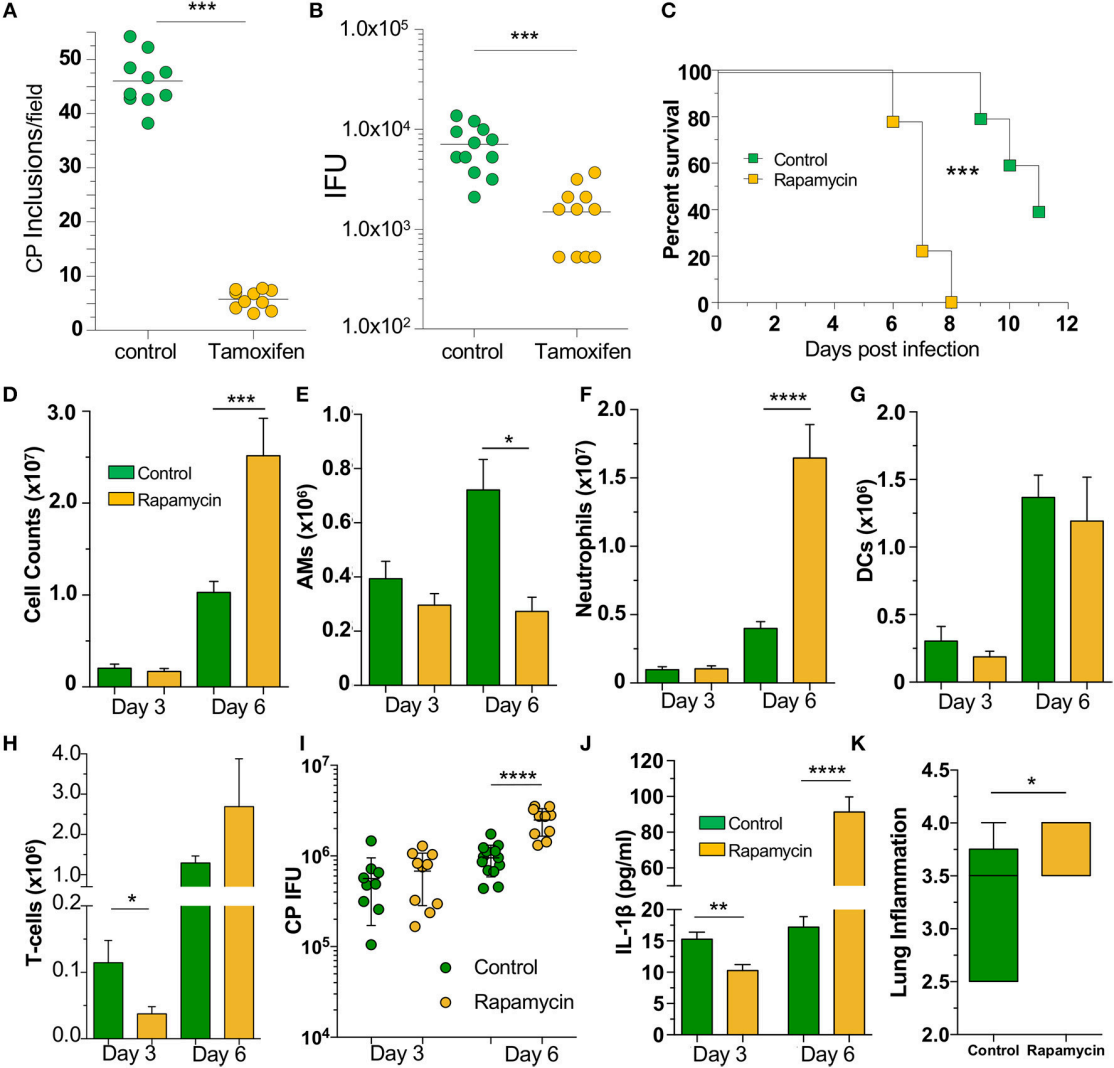
Autophagy induction fails to rescue mice from CP infection. WT MEFs were infected with CP (MOI 10) and treated with 10 μM Tamoxifen during the first 2 h of infection. **(A)** CP inclusions. **(B)** CP progeny. **(C)** C57Bl/6 mice were infected with 2 × 10^6^ IFU CP. Six hour prior to infection, mice received either sham or 3 mg/kg rapamycin i.p., and again every other day. Mortality was assessed (*n* = 5–9). **(D–J)** C57Bl/6 mice were infected with 1 × 10^6^ IFU CP. Six hour prior to infection, mice received either sham or 3 mg/kg rapamycin i.p., and again every other day (*n* = 8–12, pooled from two separate experiments). Mice were sacrificed on days 3, 6. **(D)** Total cells in the BALF. **(E)** BALF Alveolar macrophages. **(F)** BALF neutrophils. **(G)** BALF DCs. **(H)** BALF T-cells. **(I)** Bacterial burden in the lungs. **(J)** IL-1β in the BALF after CP infection and rapamycin treatment. **(K)** Lung inflammation score on day 6. Significance was assessed by students *t*-test. Survival curves was compared using the Log-Rank (Mantel-Cox) Test. Data are mean ± SEM. ^*^p < 0.05, ^**^p < 0.01, ^***^p < 0.001, ^****^p < 0.0001.

Since our data indicated that loss of autophagy in macrophages led to increased CP growth due to reduced bacterial killing, we asked whether induction of autophagy by chemical means would lead to reduced CP inclusion formation. We therefore treated WT MEFs with tamoxifen, a potent autophagy inducer (34), for the first 2 h of CP infection, and found that autophagy induction led to a significant reduction in CP inclusion formation, as well as progeny (Figures 5A, B). We next assessed whether induction of autophagy *in vivo* could rescue mice infected with a high dose of CP. Mice were i.p., injected with the autophagy inducer rapamycin (35), 6 h prior to CP infection, and every other day through the duration of the study. Surprisingly, the mice receiving rapamycin perished significantly earlier than control mice (Figure 5C). In order to determine why the rapamycin-treated mice succumbed to infection more quickly, we repeated the study with a lower infective dose and sacrificed mice 3 and 6 days after infection. Three days after infection we observed a reduction in T-cells in the BALF of rapamycin treated mice, but no changes in DCs, AMs, or neutrophils (Figures 5D–H). In contrast, while there was a decrease in the number of AMs 6 days after infection, there was a significant overall increase in cell counts in the airways primarily due to a large increase in the number of neutrophils (Figures 5D–H) in the rapamycin-treated animals. There was no difference in the bacterial burden at day 3 between control and rapamycin-treated mice, but there was a significantly greater CP load in the lungs of rapamycin-treated mice 6 days after infection (Figure 5I). Since autophagy induction can limit inflammasome activation, we measured IL-1β in the BALF and found a significant decrease in rapamycin-treated mice compared with controls 3 days after infection (Figure 5J). However, at 6 days after infection we found a significant increase in IL-1β production. We also found a clear increase in inflammation in the lungs 6 days after infection (Figure 5K, Supplemental Figure 4), correlating with the increase in neutrophils and IL-1β in the BALF, as well as the greater bacterial burden. We did not observe a difference in lung pathology 3 days after infection (data not shown). Taken together, these data indicate that the rapamycin-treated mice had greater mortality due to increased inflammation as a consequence of the greater bacterial load, likely as a result of the initial reduction in IL-1β production.

## Discussion

Our data investigating the role of autophagy during CP infection highlights the delicate balance in biological systems. This is not surprising considering the pathways investigated here and their relative importance toward immune responses. While our *in vitro* experiments demonstrate the key role that autophagy plays in controlling CP inclusion formation and bacterial growth, the overall balance *in vivo* is much more complex. Loss of autophagy in myeloid cells led to increased mortality during CP infection, but not due to increased bacterial burden. Instead, we observed a sustained increase in neutrophil recruitment to the lungs, with a corresponding increase in neutrophilic chemokines. Importantly, this was accompanied by an increase in inflammasome positive macrophages in the lung and greater IL-1β production. This sustained excess inflammation was the likely driver of excess mortality. While induction of autophagy *in vitro* limited CP growth, rapamycin treatment *in vivo* during CP infection increased mortality of WT mice, likely due to the reduction in early IL-1β production leading to an increase in bacterial burden, followed by increased IL-1β and neutrophil recruitment. IL-1β is a critical cytokine in controlling CP infection (33), yet can also be a powerful driver of pathology (36). Thus, since autophagy antagonizes inflammasome activation, any perturbation of these pathways during CP infection seems to result in a worsened outcome, creating a Goldilocks situation in which the level of autophagy and inflammasome activation must be perfectly balanced to eliminate the infection.

Common to both scenarios in which CP infected mice had reduced survival was an increase in neutrophil recruitment. CXCL1 and CXCL2 were both elevated in infected *Atg16l1*^*fl*/*fl*^ *Lysm*^*cre*^ mice, likely as a result of increased IL-1β signaling (37). Pulmonary neutrophilia is often associated with pathology and more recent studies have found that neutrophil extracellular traps can also lead to enhanced tissue damage (38). While we did not assess this in our model, this could be a mechanism by which pulmonary damage leads to decreased survival. We also observed an increase in DC recruitment to the lungs in CP infected *Atg16l1*^*fl*/*fl*^ *Lysm*^*cre*^ mice, but this did not lead to an increased T-cell presence in the lung.

The process of recycling cellular components and removal of damaged organelles has been found to play critical roles in a wide variety of diseases and cellular responses. Importantly, in the setting of bacterial infections, autophagy can be either beneficial and detrimental to the host. In multiple infections, such as *S. typhimurium, Pseudomonas aeruginosa*, or *M. tuberculosis*, loss of autophagy led to reduced bacterial clearance and subsequent enhanced pathology (13, 39, 40). In contrast, we showed that despite a clear role for autophagy in limiting CP growth *in vitro*, loss of autophagy *in vivo* did not impair bacterial clearance. This difference is likely due to the enhanced IL-1β production observed in CP-infected autophagy impaired mice, as CP is exquisitely sensitive to IL-1β (33).

While the development of novel antibiotics is one approach to combating the ever increasing number of resistant strains of bacteria, other mechanisms of enhancing bacterial clearance have also been investigated. Given that, in many cases, autophagy induction can help cells eliminate intracellular infections, several studies have investigated the antibacterial potential of autophagy induction. Supporting this effect, it has been shown that rapamycin given prophylactically in the drinking water before *P. aeruginosa* lung infection significantly reduced the bacterial burden (41), and that rapamycin treatment given i.p., reduced *Leishmania* parasite load (42). However, we found here that rapamycin treatment during CP infection actually increased CP burden and mortality. One possibility for these differing results may be the particular sensitivity of CP to IL-1β, such that the rapamycin-induced initial reduction in IL-1β resulted in enhanced CP overgrowth followed by subsequent immune-derived pathology. Perhaps a selective autophagy inducer that did not affect mitophagy and inflammasome activation might have a more beneficial outcome. Additionally, rapamycin is known to suppress T-cell responses and neutrophil extracellular traps (43, 44), which could also impair bacterial clearance in this model. Regardless, these investigations make clear that the antibacterial effects of autophagy induction are context- and pathogen-dependent.

Activation of the NLRP3 inflammasome involves damage to the mitochondria in the presence of NF-κ**B** signaling (6, 45). Damaged mitochondria are sensed by various cellular pathways and mitophagy is induced to clear them. This, in effect, leads to a reduction in inflammasome activity, and thus the manipulation of mitophagy with inducers and inhibitors directly affects inflammasome activity and IL-1β production (7, 46). We observed that disrupted autophagy in macrophages led to enhanced IL-1β secretion by CP infected macrophages *in vitro* and more IL-1β found in BALF of CP-infected mice *in vivo*. ATG7 deficiency in mice has similarly been shown to lead to increased IL-1β production during *P. aeruginosa* infection (47). However, in contrast to CP infection, autophagy disruption resulted in increased bacterial CFU in that study, corroborating earlier work showing that loss of IL-1β leads to a reduction in bacterial burden and that enhanced inflammation is beneficial to *P. aeruginosa* growth in the lung (48). In contrast, the loss of autophagy may lead to excessive IL-1β production and enhanced pathology (36), as in our CP study.

In many instances, intracellular bacterial infections manipulate or require components of the autophagy machinery to successfully infect host cells. After invading host cells, *Staphylococcus aureus* grows in double membraned autophagosomes which do not fuse with the lysosomal machinery (20). Similarly, *Yersinia pestis* can prevent acidification of the autophagosome, thereby preventing xenophagy (16). In other instances, bacteria can utilize specific components of the autophagy machinery to form the intracellular niche in which they can replicate. For example, *Brucella abortus* requires ATG14L, Beclin 1, and ULK1 to form the vacuole where it replicates (49). However, while CP grows inside a protected inclusion, loss of either ATG16L1 or ATG5 did not affect CP growth and instead led to enhanced inclusion formation and CP replication. However, as ATG16L1 and ATG5 are part of the same elongation complex in autophagosome development, it is possible that other parts of the autophagy pathway are required for CP inclusion formation.

In this study we investigated the role of autophagy during a pulmonary CP infection. While impairing autophagy limited intracellular CP growth *in vitro, in vivo* studies demonstrated that loss of autophagy in myeloid cells increased mortality, likely due to the complex antagonistic interplay between the inflammasome and autophagy. All these data suggest that, at least for some infections such as CP, any perturbation to the delicate balance between inflammasome activation and IL-1β secretion can have negative results. However, given the highly distinctive nature of bacterial pathogens, understanding each infection separately is required to fully appreciate how modulating the system would affect the end result.

## Ethics Statement

All experiments were conducted according to Cedars-Sinai Medical Center Institutional Animal Care and Use Committee (IACUC) guidelines (IACUC# 8314).

## Author Contributions

TC conceived and led the project and wrote the manuscript. TC, RP, JD, and GT performed all of the experiments. AS and EP provided critical materials to this project. SC, KS, and MA provided critical editing and content to the manuscript as well as experimental design. MA supervised the study. All of the authors read and approved the final manuscript.

### Conflict of Interest Statement

The authors declare that the research was conducted in the absence of any commercial or financial relationships that could be construed as a potential conflict of interest.
