# Supplementary material for: Autophagy Limits Inflammasome During Chlamydia pneumoniae Infection
Source: Front Immunol. 2019 Apr 12;10:754. doi: 10.3389/fimmu.2019.00754 (PMC6473188; doi:10.3389/fimmu.2019.00754)
Supplement: Supplementary file 1 [file Image_1.pdf]

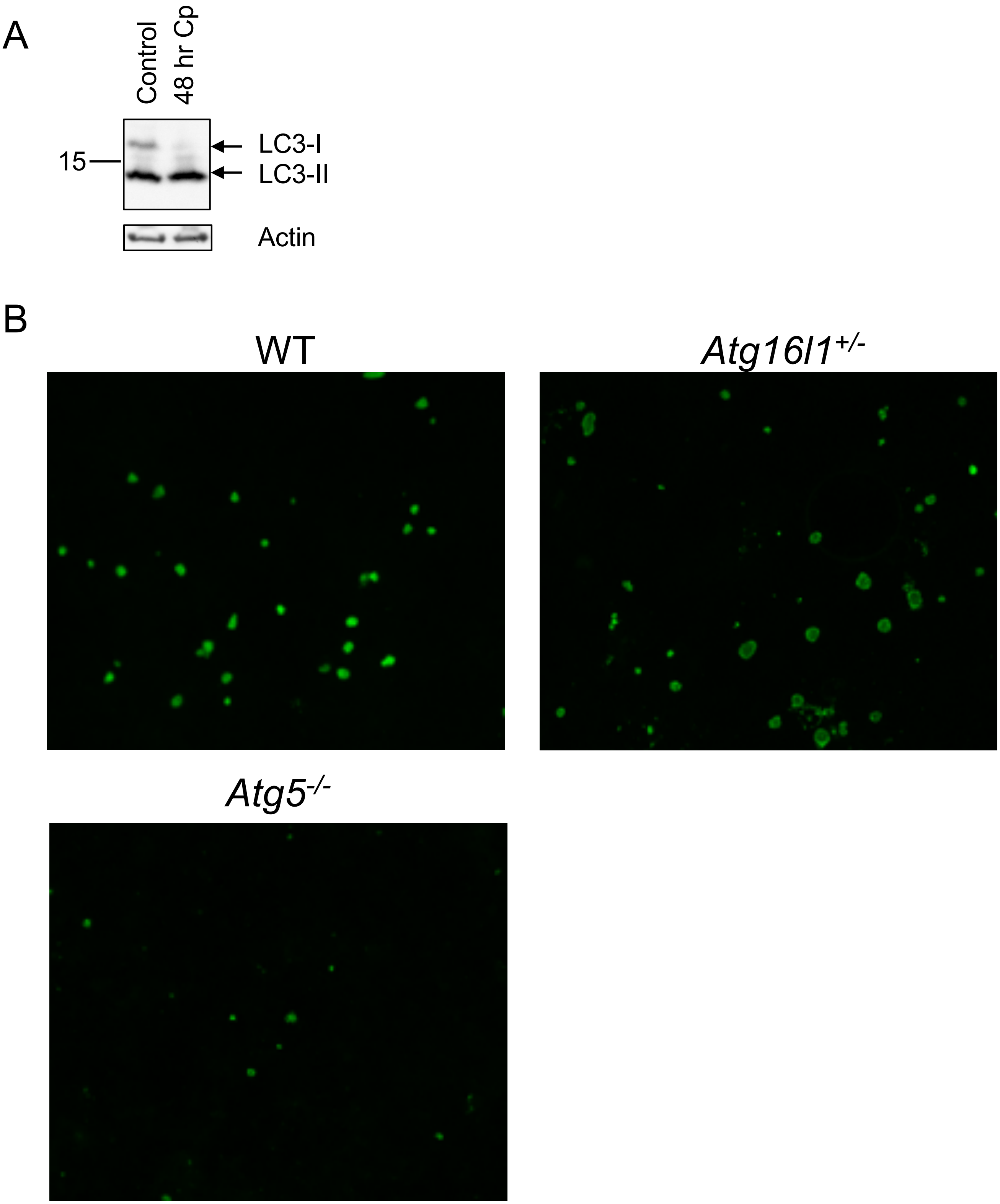

Supplemental Figure 1. Autophagy plays a role during CP growth. A) Western analysis of LC3 in WT MEFs during CP infection. B) Representative images of CP inclusions in WT, *Atg16l1*$^{+/-}$, and *Atg5*$^{-/-}$ MEFs.

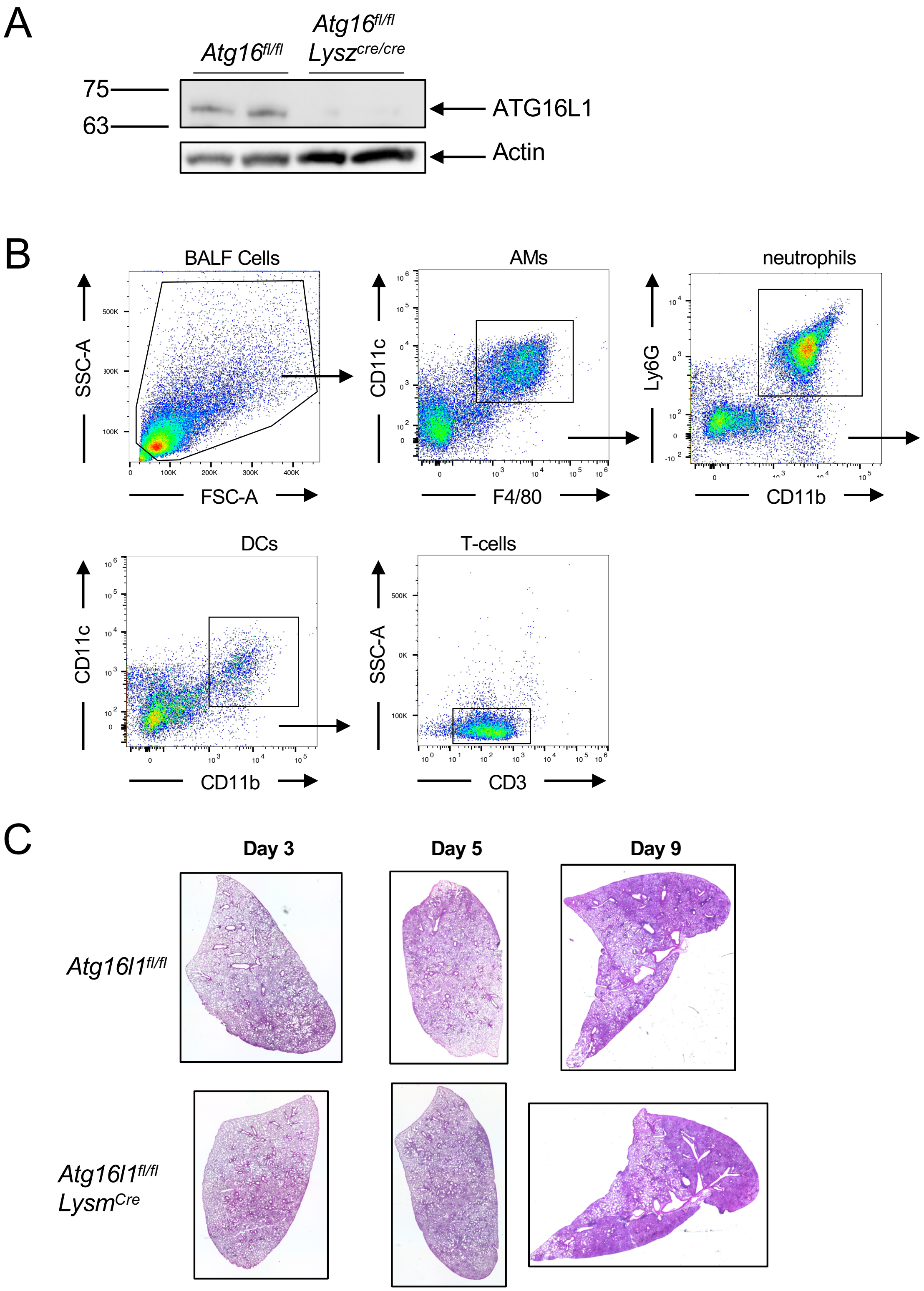

Supplemental Figure 2. A) ATG16L1 Western blot analysis of bone marrow derived macrophages. B) Gating strategy for flow cytometry analysis of BALF samples. Initial gate is positive. Subsequent gating is negative. C) *Atg16l1*$^{fl/fl}$ and *Atg16l1*$^{fl/fl}$ *Lysm*^{cre} mice were infected with 1.0x10^6 IFU CP i.t. and mice were sacrificed 3, 5, and 9 days after infection. Representative Hematoxylin and Eosin stained lung sections.

Supp. Fig 2

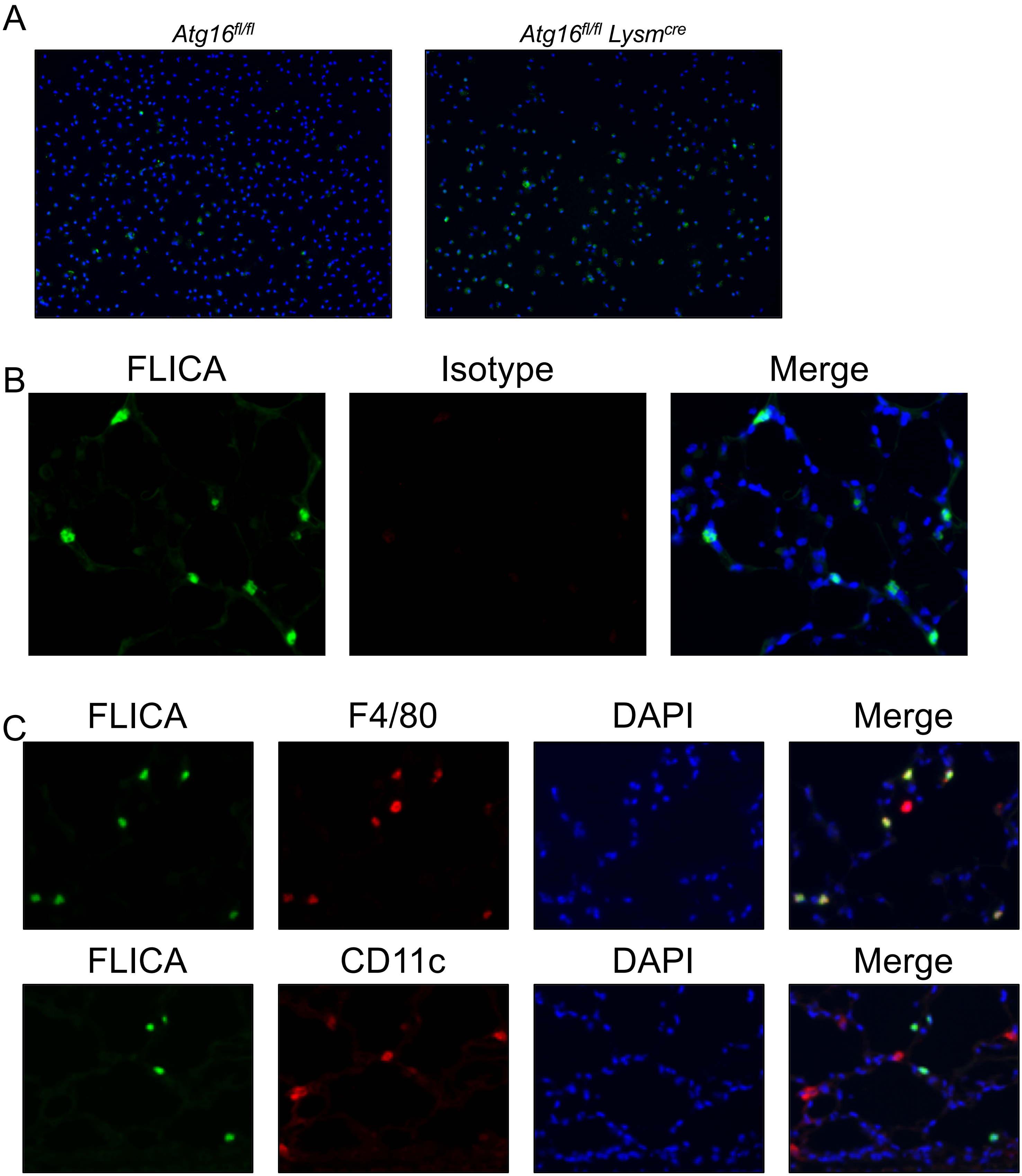

Supplemental Figure 3. Loss of autophagy in myeloid cells leads to an increase in inflammasome active macrophages during CP infection. A) Representative image of Caspase-1 activity measured by FLICA in peritoneal macrophages infected with CP (20 hr post infection). B) Isotype controls for anti-F4/80 and anti-CD11c staining. C) Caspase-1 activity measured by FLICA in frozen lung sections 1 day after CP infection. CD11c staining does not overlap with FLICA.

Supp Figure 3

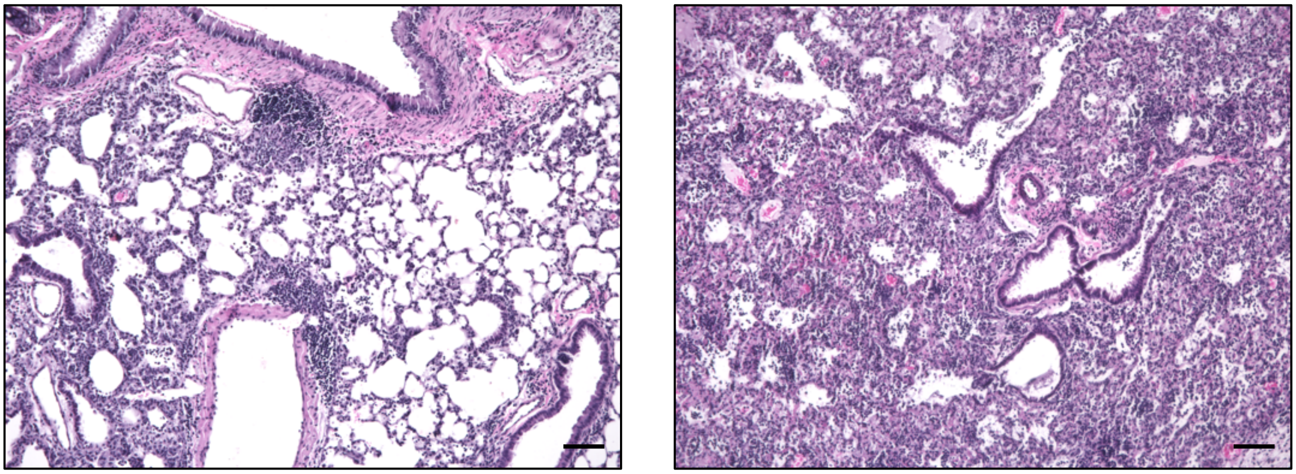

Control | Rapamycin

Supplemental Figure 4. C57Bl/6 mice were infected with $1x10^6$ IFU CP. 6 hr prior to infection, mice received either vehicle or 3 mg/kg rapamycin i.p., and again every other day (n=8-12). Mice were sacrificed on day 6. Representative H&E is shown. Scale = 100 µm.
